# Liver regeneration in aged mice: new insights

**DOI:** 10.18632/aging.101524

**Published:** 2018-08-28

**Authors:** Monica Pibiri

**Affiliations:** 1 Department of Biomedical Sciences, Oncology and Molecular Pathology Unit, University of Cagliari, 09124 Cagliari, Italy

**Keywords:** ageing, 2/3 PH, BubR1, YAP, SIRT-1, HSCs, SASP, autophagy

## Abstract

The regenerative capacity of the liver after resection is reduced with aging. Recent studies on rodents revealed that both intracellular and extracellular factors are involved in the impairment of liver mass recovery during aging. Among the intracellular factors, age-dependent decrease of BubR1 (budding uninhibited by benzimidazole-related 1), YAP (Yes-associated protein) and SIRT1 (Sirtuin-1) have been associated to dampening of tissue reconstitution and inhibition of cell cycle genes following partial hepatectomy. Extra-cellular factors, such as age-dependent changes in hepatic stellate cells affect liver regeneration through inhibition of progenitor cells and reduction of liver perfusion. Furthermore, chronic release of pro-inflammatory proteins by senescent cells (SASP) affects cell proliferation suggesting that senescent cell clearance might improve tissue regeneration. Accordingly, young plasma restores liver regeneration in aged animals through autophagy re-establishment. This review will discuss how intracellular and extracellular factors cooperate to guarantee a proper liver regeneration and the possible causes of its impairment during aging. The possibility that an improvement of the liver regenerative capacity in elderly might be achieved through elimination of senescent cells *via* autophagy or by administration of direct mitogenic agents devoid of cytotoxicity will also be entertained.

## Introduction

Although adult hepatocytes are characterized by a very low replicative rate, they can rapidly re-entry into the cell cycle following tissue loss/death [1–4]. The best characterized experimental model to study liver regeneration consists of removal of 2/3 of hepatic parenchyma (2/3 PH) in rodents [5]. In response to 2/3 PH, the remnant liver cells proliferate until the tissue mass is recovered (within 7 to 10 days). The precise molecular mechanisms associated to G0/ to G1 transition are not completely understood. However, the earliest documented changes after 2/3 PH involve increased activity of urokinase plasminogen activator (uPA) and migration of Notch1 and β- catenin to hepatocyte nuclei within 15-20 minutes [2,6]. Then, uPA-mediated extracellular matrix remodeling determines release in blood circulation of active hepatocyte growth factor (HGF), embedded in ECM in an inactive form [7–9]. Within 30-60 minutes post 2/3 PH, activation of HGF and epidermal growth factor (EGF) receptors occurs [10]. Combined EGFR and MET signaling has been found fundamental to normal hepatocyte function and liver regeneration [11]. Accordingly, their simultaneous elimination affects both the processes [11]. Furthermore, soon after 2/3 PH, a rapid increase in blood concentration of norepinephrine, tumor necrosis factor (TNF)-α, interleukin (IL) 6, serotonin, and bile acids take place [1–3,12–15]. Though no directly mitogenic, these factors orchestrate and optimize the timing and intensity of intracellular signals essential for controlling hepatocyte proliferation and paracrine cell interactions [6]. Prior to DNA synthesis, activation and nuclear translocation of transcription factors, such as signal transducer and activator of transcription 3 (STAT3), CCAAT/enhancer-binding protein beta (C/EBPβ) and nuclear factor kappa B (NFκB), occurs [2]. Enhanced expression of cell cycle inhibitors (p21 and p53), immediate early genes (IEGs) (*c-Fos, c-Jun* and *c-Myc)* transforming growth factor (TGF)-β [1] and of the transcription factors Octamer 4 and Nanog [16] are also observed. All these events lead to transcription of delayed early genes, coding for cell cycle regulatory proteins, namely cyclins [17–19]. microRNAs have also been involved in the regulation of hepatocyte DNA synthesis [20]. As an example, microRNA-21 has been found to accelerate cyclin D1 translation and cell-cycle progression during mouse liver regeneration [21]. Being liver regeneration a very strictly controlled process, hepatocyte replication ceases once the liver weight/body weight ratio has regained its original values [6,22].

Based on these findings, liver regeneration has been divided into three phases: i) priming, characterized by growth factors activation and cytokine release, ii) proliferation, promoted by immediate early gene/transcription factor activation, and iii) termination, likely governed by signal transduction pathways, such as the TGF-β-TGFβR-mediated pathway [2].

Since aging affects the regenerative response of the liver after chronic tissue injury or following surgical resection [23], it represents a critical problem in aged patients with liver disease. The first studies focusing on the effect of aging on liver regeneration date back to more than 50 years ago. At that time, Bucher et al. [24] found that the regenerative response, though preserved, was considerably reduced and retarded in aged rodents after 2/3 PH. Several subsequent studies confirmed that observation [25–29]. Despite several works focused on the subject, the molecular mechanisms underlying the age-dependent impairment of the liver regenerative capacity remain elusive.

## Intra-cellular factors affecting liver regeneration in aged rodents

Several different explanations have been proposed to justify the decline of the liver regenerative potential with age. Epigenetic alterations (i.e. histone deacetylation, methylation of gene promoter sequences and chromatin remodeling) are considered to be critical as they lead to modified expression/activation/action of genes related to hepatocyte proliferation (i.e. *Foxm1b, Cdc2, c-Myc, c-Fos, c/EBP*) [27,30,31].

About 20 years ago, Wang et al. [30] showed that reduced expression of the gene coding for the Forkhead Box M1B (FoxM1B) contributed to the proliferation defects observed in aged liver. FoxM1B is an ubiquitously expressed transcription factor restricted to proliferating cells of the mouse embryo (including liver) whose expression diminishes during postnatal cellular differentiation [32]. Liver regeneration is associated to FoxMB1 reactivation prior to S phase and sustained throughout the period of hepatocyte proliferation [32]. A positive correlation between age-related cell proliferation defects and diminished expression of FoxM1B and of its cell cycle-associated target genes has been demonstrated [33–35]. In addition, Wang et al. [30] found that *FoxM1B* gene overexpression in livers of old transgenic mice induced a regenerative response that was similar to that of young mice. Restoration of the young regenerating liver phenotype was associated with increased expression of several cell cycle-related genes (i.e. *Ccnd1, Ccna2, Ccnf, Ccnb1, Ccnb2,* coding for cyclin D1, cyclin A2, cyclin F, cyclin B1, cyclin B2, respectively). Moreover, co-transfection assays showed that FoxM1B activated transcription of cyclin B1 and cyclin D1 promoters, suggesting that those genes were direct targets of the transcription factor [30]. Collectively, all these data demonstrated that FoxM1B controls the transcriptional network of liver cell proliferation-associated genes.

Few years later, Iakova et al. [27] identified the transcription factor C/EBPα as a major contributing factor in the reduced regenerative response of aged mice to PH. In their study, Iakova et al. [27] demonstrated that in aged livers C/EBPα, a strong inhibitor of cyclin-dependent kinases (cdks) highly expressed in rodent liver [36–39], formed a C/EBPα-Rb-E2F4 complex. This complex bound to and repressed the E2F-mediated transcription of the gene *c-Myc*, which plays a central role in liver regeneration [15,40]. On these bases, authors hypothesized that post PH in young livers cdk2 is detached from C/EBPα by cyclins E and A, which bound to and activated the kinase. This, in turn, phosphorylates Rb blocking the repression of the c-*myc* promoter. On the opposite, the switch of the C/EBPα activity from cdk inhibition to repression of E2F-mediated transcription prevents old livers from eliminating the C/EBP-mediated growth inhibition post-PH [27]. It was also suggested that the switch could be related to the age-dependent increase of Brm, a chromatin remodeling protein found to interact with C/EBPα in aged livers [41,27]. In this context, it should be noted that Brm and cdk2 interact with the same region of C/EBPα [38,41]. All together, these findings led Iakova and colleagues to hypothesize that Brm might replace cdk2 in old livers leading to initiation of the C/EBPα-Rb-E2F4 complex formation and to recruitment of C/EBPα in the E2F promoters [27].

An additional hypothesis was proposed by Gagliano et al. [31] who analyzed the cell cycle gene expression in the regenerating livers of young and old rats 24 hours after CCl4 administration. They found that in aging livers the regenerative response was associated to a reduction of *c-Fos*, *c-Myc*, *Transforming growth factor-a (TGF-α)* and *Heat shock protein 70 (HSP70)* mRNA levels, while *Hgf* mRNA levels were found to be increased [42,43]. However, several controversial data on the expression profiles of cell cycle related genes in regenerating livers of young and aged mice have been reported. Indeed, while Enkhbold et al. [44], found decreased *Hgf, Met, Ccnd1* and *Ccna2* mRNA levels in aged compared to young mouse livers, more recently Pibiri et al. [45] did not observe major age-dependent changes in the expression of IEGs (c-*Jun, c-Fos* and *Egr-1*), genes coding for cytokines/growth factors (*Tnf-α,Il-6, Hgf, Tgf-α*) or transcription factors (*NF-κB, Stat3 and AP-1*) [3,21,22]. Nevertheless, in the same study *Ccnd1* gene, coding for the G1-phase protein cyclin D1, was found to be significantly down-regulated in the liver of aged mice.

Overall, the role of the early events involved in cell cycle regulation and impairment of liver regeneration in aged hepatocytes still remains elusive.

Recent studies focusing on the liver regenerative capacity of aged animals have underlined a fundamental role for intra-cellular factors involved in cell adhesion and in circadian genes regulation.

### Role of factors involved in cell adhesion: BubR1 and YAP

### *BubR1 protein regulates the spindle assembly checkpoint and is involved in cellular senescence*

Reduction in the hepatic levels of the mitotic checkpoint protein BubR1 (budding uninhibited by benzimidazole-related 1) has been suggested to be crucial in the age-dependent impairment of liver regeneration [46].

BubR1 plays an important role in the spindle assembly checkpoint to prevent chromosome unequal segregation during mitosis, thus preserving chromosomal stability [47]. BubR controls the anaphase-promoting complex, or cyclosome, (APC), a large E3 ubiquitin ligase, until all kinetochores are suitably attached to microtubules. When all the kinetochores establish bipolar attachment, APC degrades securin which, in turn, activates the separase. Then, proteolysis of the cohesion complex by separase triggers the onset of anaphase [47,48].

Several studies have also suggested a role for BubR1 in regulating aging. Indeed, decreased BubR1 expression causes cellular senescence through up-regulation of the cell cycle inhibitor p16INK4a. Moreover, mutant mice with decreased BubR1 expression (10% of the normal level) display various progeroid phenotypes [49–53].

### *Low-BubR1-expressing mutant (BubR1L/L) mice display delayed liver regeneration following PH*

Ikawa-Yoshida and colleagues [46] analyzed liver regeneration after 2/3 PH in Low*-BubR1*-expressing mutant (*BubR1L/L*) mice characterized by a 20% BubR1 reduction. These mice do not display any severe phenotype during growth and development [53], therefore providing a useful model to investigate the role of BubR1 in response to various kinds of stress. Analysis of *BubR1* gene expression in intact livers of C57Bl/6JJcl normal mice revealed a significant decrease of *BubR1* mRNA levels in aged compared to young livers [46], confirming the inverse correlation between aging and BubR1 expression. Furthermore, in *BubR1+/+* wild type animals mRNA levels of *BubR1* significantly increased post-PH, suggesting the involvement of the protein in the regenerative response. Accordingly, *BubR1L/L* mice displayed a delayed liver regeneration post-PH which was associated to increased hepatocellular necrosis and intercalated disc anomalies. The observed structural intercellular alterations consisted of widened inter-hepatocyte and peri-sinusoidal spaces, smaller hepatocytes and early-stage microvilli atrophy. These changes were associated to the BubR1-dependent reduction of the expression of desmocollin-1 (DSC1), a desmosome transmembrane cell adhesion protein, highly expressed in the liver [54].

Based on these findings, the authors proposed the following key role for BubR1 in regulating microstructural adaptation during liver regeneration. To prevent an excessive detachment-induced cell death (DICD), due to portal hypertension consequent to vasculature reduction following PH [55], only a transient DICD associated to G1 arrest occurs. The increased BubR1-mediated DSC1 expression prevents massive DICD and anoikisis, and, thus, hepatocyte focal necrosis. Indeed, DSC1 causes desmosomes reinforcement to maintain proper cell attachments. On the opposite, in conditions associated to low BubR1 expression, reduction of DSC1 is responsible for the weakened microstructural adaptation post-surgery, thus leading to hepatocyte focal necrosis. Support to these findings stems from the work of Collins et al. [56] who demonstrated that impaired cell attachment caused DICD and G1 arrest in mammary epithelial cells in a p21-dependent manner. In keeping with these results, Ikawa-Yoshida and al [46]. suggested that the delayed liver regeneration observed in (*BubR1L/L*) mice was related to p21 up-regulation, rather than to a direct effect of low BubR1 expression.

Summarizing, findings here reported attributed the decline of the regenerative response in aged liver to BubR1 reduction which leads to decreased expression of DISC1, a cell adhesion protein involved in tissue reconstitution after damage. Yes-associated protein (YAP).

### *Yes-associated protein (YAP)*

Interestingly, BubR1 expression has been found to be up-regulated by YAP (57), a downstream effector of a complex network of proteins named the mammalian tumor suppressor Hippo signaling pathway [58,59]. Hippo pathway controls organ size *via* regulation of cellular proliferation, survival and differentiation. These actions are mediated by Hippo-dependent inactivating phosphorylation and cytoplasmic retention of the transcriptional co-activators YAP (Yes-associated protein) and TAZ (transcriptional co-activator with PDZ binding motif). The most relevant residues that keep YAP and TAZ inhibited are represented by serine (S)127 and S381 in YAP and S89 and S311 in TAZ [60]. In case of loss of Hippo function, YAP and TAZ move to the cell nucleus and associate with various DNA-binding proteins, e.g. TEAD factors, driving gene transcription [61,62]. In rodent livers YAP is involved in proliferative signals, and its increased expression causes hepatomegaly and, when sustained, HCC development [62–65].

### *YAP protein controls transcription of spindle checkpoint genes through physical association with BubR1*

Recent studies of Yang et al. [66,57] have shown that in mammalian epithelial cells the mitotic phosphorylation of YAP by CDK1, mainly in tyrosine (T)119 and S289 residues, ensured the spindle check point activation, responsible for correct chromosome segregation and mitosis. Furthermore, overexpression of mitotic phosphorylated YAP determined dysregulation of the spindle checkpoint, failure in maintaining normal mitosis and genomic integrity, and oncogenesis [66,57]. Mechanistically, the Authors have documented that the mitotic phosphorylated form of YAP controls transcription of spindle checkpoint genes through physical association with BubR1 [57]. Notably, mitotic phosphorylation of YAP was also required for BubR1 transcription [57]. Thus, BubR1 was suggested to be a transcriptional target of YAP in mediating spindle checkpoint function.

Given the role of BubR1 in regulating cell adhesion and liver regeneration [46], its YAP-dependent transcriptional activation appears very interesting. Indeed, similarly to BubR1, YAP is involved in cytoskeleton and cell adhesion regulation [67] and its reduced activation has been correlated to liver regeneration impairment during aging [68,45].

### *YAP protein is regulated by mechanical signals*

Several studies have documented YAP involvement in mechanical signaling. Indeed, cell density [69], cell adhesion [69], cell morphology [70], ECM rigidity [71], and mechanical cell stress [72] have been identified as regulators of YAP/TAZ activity [67,73,74]. Support to this notion stems from the following findings: i) in low cell density culture YAP is predominantly localized to the nucleus in its active un-phosphorylated form while in high density culture is phosphorylated and localized in the cytoplasm [69]; ii) in cells grown on small domains YAP is mostly cytoplasmic, whereas it is localized in the nucleus on large domains [70]; iii) on hard substrates, YAP and TAZ are predominantly nuclear while increasingly accumulate in the cytoplasm on softer substrates [71]; iv) mechanical stretching of contact inhibited cells induces YAP/TAZ re-entry into the nucleus to stimulate cellular proliferation [72].

Collectively, all these studies have identified actin cytoskeletal reorganization as a dominant regulator of YAP and TAZ activity [73]. Studies suggesting a functional connection between G protein-coupled receptor (GPCR)/Rho signaling, cytoskeletal reorganization and YAP/TAZ activity support this finding [75–77]. In particular, Yu et al. [75], have reported that activation of GPCRs by chemical stimuli regulates YAP activity depending on the particular G protein coupled to the receptor. Indeed, G-protein binding may cause increase or inhibition of YAP function by enhancing or inhibiting the activity of the YAP inactivating kinase Lats1/2 [75,77]. Notably, also F-actin polymerization status and change of cell adhesion mediated by Rho GTPase deactivation and cytoskeletal reorganization were shown to be correlated with YAP activation [75,77]. In general, from these studies, it emerges that increased Rho GTPase activity and actin polymerization activate, whereas destabilization of actin inhibit, YAP and TAZ [77].

### *High basal and post-PH levels of YAP are counteracted by high levels of YAP inactivating kinases in aged livers*

As to the role of YAP during liver regeneration post-PH, Pibiri et al. [45] found that while YAP protein expression essentially reflected progression into cell cycle in young mice, an early, robust and persistent increase of the protein occurred in aged mice. In spite of YAP increase, liver regeneration resulted reduced in aged animals. According to the Authors, the persistent increased levels of YAP in aged liver represents a signal that senses the decreased liver mass, and consequently, it is aimed at stimulating a regenerative response in order to re-establish the original liver size [45]. More recently, Laforese et al. [68] found that aged mouse livers had higher basal levels of YAP than young ones. Interestingly, aged animals also showed higher basal and post PH levels of the active form of the YAP inactivating kinases MST and LATS [68] compared to young mice. Furthermore, siRNA-mediated silencing of MST1 and MST2 was able to induce hepatocyte proliferation in quiescent young livers and to rescue regeneration in aged ones following PH [68]. These findings suggest a direct link between YAP activation and liver regeneration. Nevertheless, the involvement of MST1 in diverse signaling pathways [78–80], does not rule out the possibility that cooperation of YAP with other effectors is also involved in liver regeneration.

### *Cooperation between YAP and BubR1 in mediating liver regeneration: a possible scenario*

**Figure 1:**
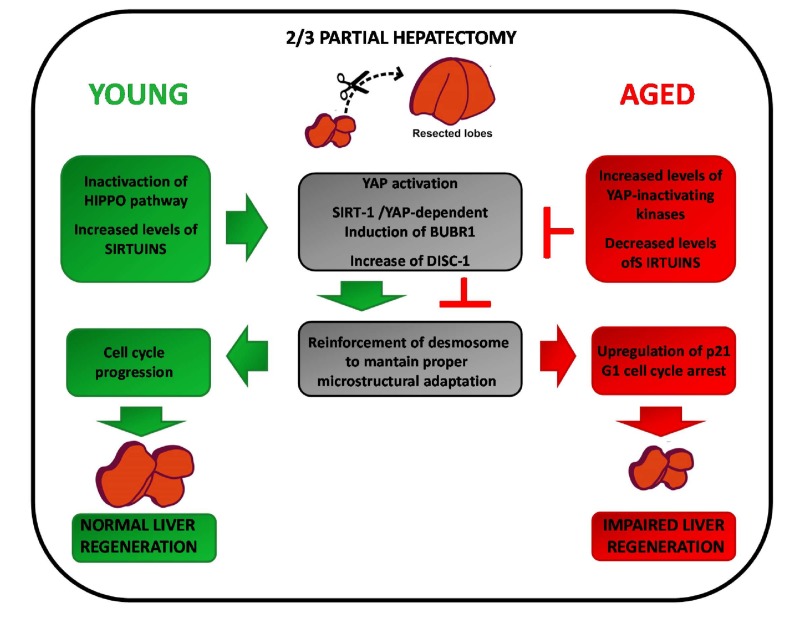
**Intracellular factors affecting compensatory regeneration in aged livers.** *Young livers (left):* reduction of the liver mass after PH leads to YAP/Sirtuin-dependent transcription of *BubR1*. BubR1, in turn, induces the cell adhesion protein DISC1 which provides the proper microstructural adaptation during regeneration. *Aged livers (right):* the increased levels of MSTs counteract YAP activation. Furthermore, as aging is associated to decreased SIRT-1 expression, there is a reduction of the YAP/Sirtuin-dependent transcription of *BubR1*. This lead to reduction of DSC1 expression, weakened microstructural adaptation and upregulation of p21. As a result, G1 cell cycle arrest and impairment of liver regeneration is observed.

Given the findings that: i) BubR1 is a direct transcriptional target of YAP [57]; ii) both YAP and BubR1 are involved in regulating cell adhesion and positively modulate cell proliferation [46,57,61,63,64,67]; iii) both BubR1 and YAP expression/activation are reduced in condition of impaired liver regeneration [45,57,68], the following scenario can be hypothesized. Under physiological condition the Hippo pathway efficiently controls the size of the liver. Indeed, cell-cell and cell-extracellular matrix contacts and high cell density activate the Hippo pathway through phosphorylation-dependent inactivation of YAP and its retention within the cytoplasm. In young mice, reduction of the liver mass after PH leads to YAP activation and subsequent transcription of BubR1 which, in turn, induces DISC1 protein, thus guaranteeing a proper micro structural adaptation during tissue regeneration. In aged livers, however, the increased basal levels of YAP inhibitory kinases, such as MSTs, counteract the positive signals consequent to loss of liver mass [68]. As a result, despite increased levels of YAP proteins, in aged hepatectomized livers the tissue mass cannot be properly restored due to the YAP inactivating phosphorylation mediated by MSTs [68]. Consequently, the decreased transcription of *BubR1*, a YAP target gene, leads to DSC1 reduction, weakened micro structural adaptation and p21 upregulation which, in turn, causes G1 cell cycle arrest and impaired liver regeneration (Fig 1). This scenario implies a coordinate action between YAP and BubR1 to rescue conditions at risk of tissue damage, such as liver regeneration after surgery or the worsening of necrosis induced by cytotoxic agents. It follows that uncoupling of their activity may impair liver regeneration in aged liver, but only in association with tissue loss. According to this view, one may explain why aged hepatocytes still retain their fully replicative capacity following exposure to direct mitogenic stimuli, which are able to induce hepatocyte proliferation in the absence of tissue damage [81].

## Role of factors affecting the circadian clock: SIRT1

### *The circadian clock*

The cellular and physiological rhythms observed over a 24 hour period are termed circadian rhythms. These regulate many physiological processes, such as sleep/awake, metabolism and hormonal secretion [82,83]. In mammals, the circadian timing system is composed of a central pacemaker, housed in the brain’s suprachiasmatic nucleus (SCN). The rhythm-generating mechanism is thought to rely on a feedback loop involving positively and negatively acting transcription factors [84–86]. More in detail, aryl hydrocarbon receptor nuclear-translocator-like (ARNTL or BMAL1) and Circadian Locomotor Output Cycles Kaput (CLOCK) transcription factors activate the expression of Period (*Per*) and Cryptochrome (*Cry*) genes, and once PER and CRY repressor proteins accumulate to a critical level they bind to BMAL1-CLOCK heterodimers and thereby repress the transcription of their own genes [84–86]. Although the master circadian clock is located within the SCN, the key circadian proteins are expressed in many peripheral tissues, determining circadian periodicity in gene expression and physiology for many organs [86]. Indeed, microarray data have shown that up to 10% of genes in different tissues are directly or indirectly regulated by the circadian clock system [87,88].

### *Aging is associated to degradation of the neuronal activity rhythms in SCN and to deregulated nutrient sensing*

It is commonly assumed that the circadian clock and the aging process are intimately entangled. More specifically, it has been demonstrated that changes in central rhythmic behavior occur with ageing [89], mainly due to the age-dependent degradation of the neuronal activity rhythms in SCN [90]. On the other hand, mice deficient in core circadian transcription factor (BMAL1) expression have reduced lifespan and premature aging phenotype [91]. Aging-associated deterioration is associated to disruption of metabolic homeostasis, including deregulated nutrient sensing [92–97]. Accordingly, caloric restriction (CR) has been found to extend lifespan in several organisms and to rewire circadian metabolism [97–102].

### *Sirtuins*

Sirtuins are a nicotinamide adenine dinucleotide (NAD+)-dependent family of histone deacetylases (HDACs) which are involved in various physiological functions, such as aging, genome integrity maintenance, stress response to nutrient challenge, metabolic control and cancer [103–105]. HDACs mediate deacetylation of histones which leads to gene silencing [106–108] but are also implicated in the reversible acetylation of non histone proteins [109–111]. The catalytic reaction mediated by sirtuins involves the breakdown of one molecule of NAD+ for each deacetylated acetyl lysine and the generation of nicotinamide and O-acetyl-ADP-ribose, which serves as an acyl acceptor to form an acylADP-ribose product. Similar to other posttranslational modifications, the presence or absence of acyl groups on specific lysine residues in proteins can determine their function and subcellular localization [112–114]. Apart from their deacetylase function, the seven mammalian members of the sirtuin family have different enzymatic activity, biological targets and cellular functions [115,116].

### *SIRT-1 contributes to the modulation of the circadian clock by nutrients and of the aging process*

SIRT-1, member of sirtuin family, has been found to deacetylate histones and several transcription factors involved in the control of metabolism [117–121], in particular following nutritional deprivation [121–123]. Recently, Orozco-Solis et al. analyzed the role played by SIRT-1 in the modulation of the SCN by nutritional inputs by using mice with *Sirt1* ablation in the steroidogenic factor 1(Sf1) neurons of the ventromedial hypothalamus (VMH) [124]. Indeed, Sf1-expressing neurons, exclusively located in the VMH, were demonstrated to be required for the regulation of energy balance and glucose metabolism [125,126]. They found that SIRT-1 in the VMH operated as a metabolic sensor able to connect food intake to circadian behavior. Indeed, under food restriction and absence of light, SIRT-1 contributed to activity behavior which was associated to changes in the acrophase and amplitude of core clock genes in the SCN. Thus, under specific physiological conditions, SIRT1 contributes to the modulation of the circadian clock by nutrients.

Recent studies have also documented a link between SIRT-1-dependent core clock gene regulation and aging [114,127]. Accordingly, it has been demonstrated that the brain-specific *Sirt1^−/−^* (BSKO) mice exhibited dampened circadian gene expression in the anterior hypothalamus (where the SCN is located). This was associated to a lengthened circadian period and an accelerated aged phenotype [128]. Thus, loss of SIRT-1 in the brain not only regulates the circadian clock but also accelerates the aging process, which is most likely mediated by NAD+. The additional findings of the mammalian SIRT-1 involvement in circadian control of liver gene expression and metabolism has documented the existence of an interplay between aging, nutritional challenge and circadian metabolism [103,129,130].

### *Caloric restriction is able to rescue the decline in rhythmic global protein acetylation of aged liver by enhanced activity of SIRT-1*

Recently, Sato et al. [131] analyzed the mechanisms associated to CR-dependent improvement of circadian rhythms. To this aim, they have paralleled the transcriptomic profile of liver cells with those of muscle and epidermal stem cells of young and old mice [132] [104], fed *ad libitum* with either normal chow diet or under caloric restriction (CR). Data obtained revealed that aging and CR caused remarkable tissue-specific circadian changes in the liver and led to the identification of a rhythmic global protein acetylation as a hallmark of the liver clock. Remarkably, this oscillatory acetylation signature was drastically affected in old mice and was rescued under CR regimen. The authors attributed changes in CR-associated protein acetylation to improvement in NAD^+^ availability, increased levels of acetate and acetyl-CoA and enhanced activity of SIRT1 [133–135]. Accordingly, an enrichment of SIRT1-target genes was found in mouse livers under CR [136]. Control of protein acetylation was shown to be mediated at least in part, by SIRT1 and the dynamic acetylation of cytoplasmic Acetyl-CoA Synthetase 1 (AceCS1). Indeed, previous studies demonstrated that SIRT-1 was able to deacetylate and control cytoplasmic AceCS1 [137], as a regulatory circadian pathway which results in the production of cyclic pools of acetate-derived acetyl-CoA [138]. Accordingly, CR was associated to increased hepatic acetate levels in young mice and extended acetylation profile of AceCS1 in young and old animals [131]. Although a causal relationship was not directly demonstrated, Authors have hypothesized that the change in circadian acetylation of AceCS1 could be linked to the increased levels of acetyl-CoA and global protein hyperacetylation observed under CR [131]. Summarizing, this study demonstrates that aging affects cyclic global protein acetylation, mediated by timely activation of both histone acetyltransferases (HATs) and HDACs, which can be rescued by nutritional deprivation.

### *Decreased SIRT-1 expression in aged liver may impair liver regeneration via reduction of BubR1*

A direct molecular link between the circadian clock and cellular proliferation has been established [139]. Indeed, rhythmic expression of key regulators of cell cycle progression and of the DNA-damage response is dependent on the circadian machinery [140–142]. Moreover, liver regeneration is impaired in mice deficient in core clock genes [143]. Based on these findings and on the role of SIRT-1 in regulating circadian genes, Bellet et al., analyzed liver regeneration following PH in liver-specific Sirt1-deficient mice [144]. Their results showed that G1/S progression, as well as circadian gene expression, were significantly affected by the loss of SIRT-1.

These results suggest that the decreased expression of SIRT-1 observed in aged liver [131] could impair liver regeneration post PH. In this context, notably, Singh et al. identified BUB family proteins, i.e.BubR1, as downstream targets of SIRT-1 in melanoma cells [145]. In particular, they demonstrated that specific inhibition of SIRT-1 by tenovin-1 was associated to decrease of both protein and mRNA levels of BuB proteins. Thus, not only decreased YAP activation, but also decreased SIRT-1 levels could cooperate to determine BubR1 reduced expression in aged livers, thereby affecting liver regeneration after tissue injury [68,144,145]. (Figure 1).

## Extra-cellular factors affecting liver regeneration in aged rodents

It was shown that aging does not affect mouse hepatocytes replicative capacity following exposure to mitogenic agents, such as nuclear receptor ligands (NRL) [81], able to induce cell proliferation in the absence of tissue injury (direct hyperplasia). The early signal transduction pathways involved in nuclear receptor-mediated hepatocyte proliferation are quite different from those of liver regeneration. Indeed, neither activation of transcription factors (AP-1, NF-κB, STAT3, and C/EBP), nor increased expression of immediate early/growth factor genes (*c-Fos, c-Jun, c-Myc*, *Lrf-1, Egr1, Hgf and Tgfα*) or release of the cytokines tumor necrosis factor *α* (TNF-α) and interleukin *6* (IL-6) could be observed in rodents after treatment with NRL [146–151]. Those results demonstrate that the intrinsic hepatocyte replicative capacity is maintained during aging if an appropriate proliferative stimulus is provided. Based on this, it has been suggested that extrinsic factors (*i.e.* growth factors, cytokines, hormones) rather than intrinsic changes within the cell could be responsible for the depressed replicative response observed in aged rodent liver [81]. Accordingly, a pioneer study of Conboy at al [152], reported that heterochronic parabiosis (a shared circulatory system between young and old mice) was able to increase proliferation of hepatic progenitor cells of aged animals. Enhanced liver cell proliferation was associated with a decrease in aged livers of the c/EBPα complex, a known cell proliferation inhibitor whose expression increases with age. This finding suggested the existence of systemic factors able to modulate the molecular signaling pathways critical to liver-specific progenitor cell activation which are present in young liver microenvironment but are lost in aged livers.

A number of extra-cellular factors has been implicated in age-dependent impairment of liver regeneration, including growth hormone (GH) [153], Src homology 2 domain-containing (Shc) protein p66^Shc^ [154] and interferon gamma (IFN-ϒ) [155].

As to GH, Moolten et al. showed that its administration to rats accelerated liver regeneration after PH [156]. Later studies by Krupczak et al. [153] found that GH administration to old mice was able to improve liver regeneration post-PH by increasing FoxM1b levels to those seen in young animals after surgery. Moreover, Jin et al. [157] demonstrated that GH improved liver regeneration through glycogen synthase kinase 3 (GSK3) regulation. In particular, they showed that in young animals, high levels of GH increased the expression of GSK3. This kinase associated to and degraded cyclin D3, an activator of cdk4 which positively regulates the growth inhibitor C/EBPα in aged mouse livers [158]. Opposite, in aged livers the GSK3 promoter was found to be repressed by C/EBP-histone deacetylase 1 (HDAC1) complexes, leading to the GSK3 reduction. Accordingly, treatment of old mice with GH increased expression of GSK3 *via* removal of the C/EBP-HDAC1 complexes from the GSK3 promoter. Furthermore, while down-regulation of GSK3 in young mice inhibited liver cell proliferation post-PH, *via* the cyclin D3-C/EBP pathway, its up-regulation in old mice accelerated hepatocyte proliferation [157]. On this basis, GSK3 has been considered as a key regulator of liver cell proliferation whose age-dependent reduction affected the liver regenerative capacities.

p66Shc protein is a member of the Src homology 2 domain-containing (Shc) A family, ubiquitously expressed except for in the brain and in neurons [154]. ShcA proteins have been found to act as adaptor molecules involved in epidermal growth factor-mediated Ras/mitogen-activated protein kinase (MAPK) cascade activation. However, p66Shc was shown to inhibit activation of the Ras/MAP kinase pathway, by competing with p46Shc and p52Shc for binding to the adaptor protein Growth factor receptor-bound protein 2 (Grb2) [154,159–162]. Moreover, it was shown to induce cell oxidative stress (OS) and apoptosis [163–165]. Accordingly, loss of p66Shc has been shown to be associated to increased resistance to OS and apoptosis [163,164] and to protection against ROS-mediated acute tissue damage [165]. Furthermore, although aging is characterized by elevated levels of ROS, p66Shc knockout mice display a higher resistance to OS and an increased lifespan compared to their wild-type counterpart [166]. Thus, p66shc has been proposed to act by monitoring the intracellular concentration of ROS in order to eliminate cells injured by oxidative damage [167]. On these premises, Haga et al. [167] decided to analyze the role of p66Shc in liver regeneration post-PH during ageing. They found that impairment of tissue recovery in aged mice was associated to OS, which was an early event post-surgery, followed by marked apoptosis. Interestingly, p66Shc was strongly phosphorylated at S36 early after PH. Notably, this phosphorylated form of p66Shc (p6636S) has been reported to induce cellular OS by suppressing the activity of the anti-oxidant/pro-survival molecule Akt and the expression of catalase [163,164,166]. Furthermore, p6636S seems to be critical for induction of apoptosis in cell exposed to OS [163,164,166]. Accordingly, while several markers of cell proliferation were expressed/activated after PH in livers of both aged and young mice, Akt was not activated in the former group [167]. Ablation of hepatic p66Shc remarkably improved liver regeneration post-surgery in aged animals, as well as Akt activation, which was associated to suppression of post-surgery OS and apoptosis. Interestingly, loss of p66Shc did not affect post-PH liver regeneration in young mice. Thus, these findings [168] suggest a key role for p66Shc in the impairment of liver regeneration during aging.

Several studies have shown an up-regulation of the inflammatory response with age across various tissues [168,169]. Accordingly, Singh et al. [170] described immune cell infiltration and elevation of the transcript levels of various growth inhibitory inflammatory cytokines, such as transforming growth factor-β (TGF-β), IFN-ϒ and interleukin (IL)-1, in the liver of aged animals.

IFN-ϒ inhibits cell cycle through activation of the cyclin-cdk complex inhibitor p21 *via* phosphorylation of the STAT1 protein, which, in turn, activates the IFN-specific transcription factor IRF1 [171]. IRF1 causes p53 nuclear accumulation [172,173] and increased nitric oxide production via inducible nitric oxide synthase induction [174,175] which, subsequently, lead to p21 transcription [176]. Based on these findings, Singh et al. analyzed the role of IFN-ϒ in the impairment of liver regeneration post-PH in aged mice [177]. They found higher IFN-ϒ levels in aged livers compared to young tissues which were associated to increased IFN-ϒ-dependent gene transcription both before and after PH. The increased IFN-ϒ levels observed in aged livers negatively correlated with liver regeneration post-surgery. *In vivo* deletion of macrophages and natural killer cells, the major IFN-ϒ producers, led to restoration of the proliferation kinetics post-PH in aged mice. Furthermore, aged IFN-ϒ^−/−^ mouse livers exhibited an earlier entry into the cell cycle compared with age-matched controls. Thus, these findings strongly suggest that the age-related increase in the pro-inflammatory status in the liver [170] has a detrimental effect on its regenerative capacity. From these studies it emerges that the age-dependent increase of oxidative stress and inflammatory molecules plays a crucial role in liver regeneration.

**Figure 2:**
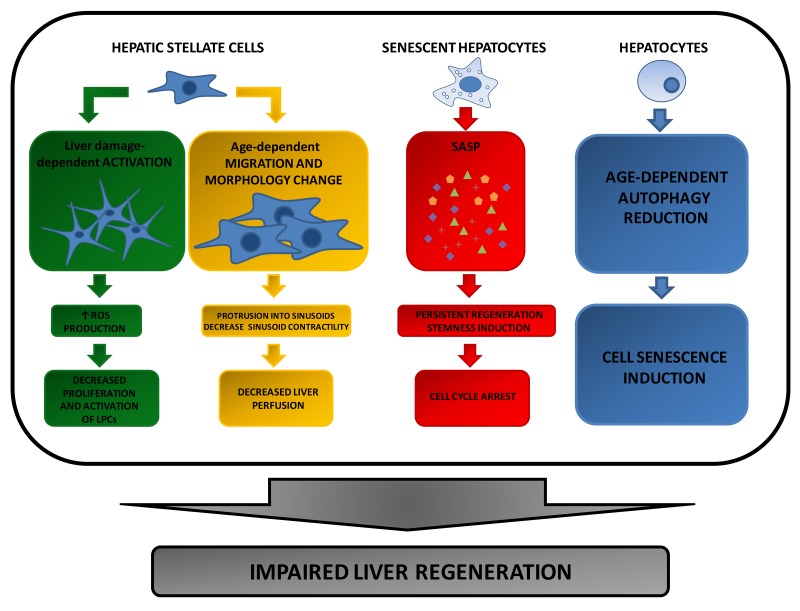
**Extracellular factors affecting compensatory regeneration in aged livers.** During ageing, activated HSC-induced chemokine production leads to a decreased activation and proliferation of Liver Progenitor Cells (LPCs). Since these cells are required to repopulate the liver following tissue injury, their decrease results in a reduced liver regenerative response. Furthermore, age-dependent HSC size increase and migration, can negatively affect hepatocyte proliferation by reducing liver perfusion. A further reason for the impaired liver regeneration of aged hepatocytes is due to the pro-inflammatory proteins chronically released by senescent hepatocytes that accumulate in the elderly as a consequence of a reduction of autophagy program.

Extracellular factors involved in the regulation of oxidative and inflammatory status also represent the focus of the most recent studies on liver regeneration during ageing (Figure 2).

### Role of factors affecting oxidative status and liver perfusion: changes in hepatic stellate cell (HSC) activity, phenotype and localization

### *HSCs mediate the negative regulation of LPCs during liver regeneration in aged animals*

Based on the free radical theory, ageing is the consequence of the accumulation of reactive oxygen species (ROS) due to free radical incomplete destruction by the appropriate endogenous defense systems [178]. While generation of ROS is essential to maintain cellular homeostasis, when excessive, it might lead to tissue damage and to activation of specific signaling pathways influencing aging and age-related disease development [179].

Recently, Cheng et al. [180] have demonstrated that oxidative stress inhibits liver progenitor cell (LPC) activation in aged mice during liver regeneration. In particular, they [180] have analyzed liver regeneration following choline devoid-ethionine supplemented (CDE) diet. This dietary regimen causes liver damage and expansion of LPCs, also known as ‘oval cells’ [181]. Upon massive liver injury, LPCs proliferate and migrate into the hepatic lobule where they can differentiate into hepatocytes and/or biliary epithelial cells [182–186]. Notably, LPC expansion occurs in many human liver diseases [187] and experimental animal models [188].

In the study of Cheng et al. [180], it was shown that CDE administration induced LPC proliferation in young mice, but not in old animals. Loss of LPC proliferation was associated to the impairment of the regenerative response of aged livers. Interestingly, no significant difference in the clonogenic and proliferative capacity of isolated LPCs was found between young and old mice. Thus, no intrinsic cellular changes can be claimed as causative factors for age-dependent reduction in liver regeneration. Accordingly, the age-dependent decrease of LPC proliferation appeared to be due to extracellular factors, in particular excessive ROS levels produced by neutrophils recruited into the cell niche by activated hepatic stellate cells (HSCs) [180] .

Summarizing, in this study HSCs are recognized as the critical cell population in the negative regulation of LPCs during liver regeneration in aged animals. Nevertheless, more recent studies questioned the role of LPCs in repopulating the liver after tissue damage [189–193]. Indeed, novel genetic lineage tracing experiments have shown that virtually all hepatocytes in the injured liver derive from self-expansion of the pre-existing hepatocytes [185,186,194,195]. These studies attributing to LPCs a negligible contribution in liver regeneration cast doubts about a critical role of HSCs in liver mass recovery. Nevertheless, HCSs have been suggested to affect liver regeneration during ageing by further mechanisms.

### *Changes in HSC phenotype and localization during aging could affect liver regeneration response via reduction of tissue perfusion*

An additional role of HSCs in the ageing process that could be related to the impairment of the liver regenerative capacity has been proposed by Marcos et al. [196]. These Authors found that during aging, HSCs migrate from the centrilobular to the periportal areas where they display a modified morphology characterized by cell enlargement due to increased content of lipid droplets. In particular, during ageing HSCs morphology changes from a small cell body with long and thin extensions to a large cell body with thicker and much shorter extensions. A larger cell body of HSCs has been implicated in the blood flow reduction observed in the sinusoids of older animals [197], due to cell protrusion into sinusoids [198]. Marcos et al. suggested that also the age-dependent shortening of HSC processes could play a role in liver flow reduction [196]. Indeed, the HSC processes have been shown to exert a pivotal role in sinusoid contractility acting as chemotactic signal sensors [199]. Accordingly, it has been hypothesized that the shorter processes observed in aged HSCs could surround and control the blood flow of fewer sinusoids than in young mice where HSCs encircled more than two sinusoids [200]. Even if the study of Marcos et al. [196] was not focused on liver regeneration, it provides another possible explanation for the impaired liver regeneration observed in the elderly, as already suggested by Saito et al. [201].

## Role of factors affecting inflammation status: SASP

### *Cellular senescence and “senescence-associated secretory phenotype” (SASP)*

Chronic inflammation is associated to normal aging and to several age-related diseases, such as cancer, atherosclerosis and osteoarthritis [202,203]. While in the past it has been mainly attributed to the progressive activation of immune cells over time [202,204,205], recent studies have proposed that cellular senescence might be an important additional contributor to chronic inflammation [206–208].

Cellular senescence is thought to be a tumor suppressive stress response [209–211] which is also associated with aging. Indeed, senescent cells accumulate with ageing and a variable proportion of them, interspersed in islands of normal cells, is a common feature in aging mammalian tissues [212–215].

Wang et al. [212] have examined the process of hepatocyte senescence in normal mouse liver during a time period of 18 months. They have found that the percentage of hepatocytes characterized by polyploidy, accumulation of DNA-damage and activation of p21 and p16ink4, all common features of cell senescence, increased with age [212]. This accumulation of cell cycle arrested cells leads to the age-dependent loss of functional and regenerative tissue capacity which prevents proliferation of somatic and stem cells in a cell intrinsic-manner [216–222]. Common age-dependent cell intrinsic defects comprehend p53 activation and increased expression of the cyclin-dependent kinase inhibitor p16 INK4a [216–222].

Similar to mouse hepatocytes, human hepatocytes undergo age-dependent senescence [212,223] and it has been shown that poor liver regeneration in older patients was associated with up regulation of senescence-related genes, such as *p16*, and down-regulation of regeneration-promoting ones, such as *HGF* and *MET* [224].

Senescent cells have been demonstrated to disrupt tissue structure and function through secretion of pro-inflammatory factors participating in intercellular signaling [206–208,225]. Indeed, despite of an apparently irreversible growth arrest, these cells are metabolically active and are characterized by a peculiar morphology, physiology and gene expression [225]. Secretion of pro-inflammatory factors has been termed as “senescence-associated secretory phenotype”, or SASP, and it is generally induced at transcriptional level [206]. SASP includes a wide range of growth factors, proteases, chemokines and cytokines (*i.e.* pro-inflammatory proteins IL-6, IL-8, IL-1, granulocyte macrophage colony stimulating factor (GM-CSF), growth regulated oncogene (GRO)α, monocyte chemotactic protein (MCP)-2, MCP-3, MMP-1, MMP-3, and many of the Insulin-like growth factor (IGF)-binding proteins) [206,226–228]. In addition, to reinforce the growth arrest and induce senescence in a paracrine manner [222,225,229], SASP has also been shown to favor wound healing [230], embryonic development [231,232] and tumor growth [233,234], suggesting more complex physiological roles than currently understood.

### *SASP could affect liver regeneration during aging by blocking neighboring cells in a stem like state*

A possible explanation to the apparent discrepancy between the growth inhibitory and tumor suppressive effect of senescence and the tumor promotion activity of SASP, could be found in the recent study of Ritschka et al. [235]. Briefly, these Authors have shown that transient exposure of mouse primary keratinocytes to SASP determined increased expression of stem cell markers and regenerative capacity *in vivo*. On the opposite, a prolonged exposure caused a subsequent increased stemness which was blocked by a cell-intrinsic senescence arrest mediated by the cell cycle inhibitor p19. These results were interpreted to suggest that cells sensed a prolonged SASP-induced regeneration as a pro-tumorigenic event, and, therefore, activated cell-intrinsic tumor-suppressive mechanisms as a protection devise. Furthermore, in the same study, it was demonstrated that senescence induction in single liver cells *in vivo* was associated to activation of tissue-specific expression of stem cell markers. This has supported the finding that SASP can induce stemness in the surrounding tissue in a paracrine manner [235]. According to the Authors, the discovery that timely controlled exposure to SASP can directly promote tissue regeneration identifies new important biological roles for the senescence program, supporting the idea that senescence might be primarily a beneficial and pro-regenerative process. However, when perturbed, it can result in tumor formation and aging.

More recently, de Keizer et al. [236] have hypothesized that the SASP ability to trigger reprogramming of neighboring cells into more pluripotent cells *in vivo* [235,237] could be responsible for the reduced tissue regeneration capacity during aging. Indeed, they have proposed that in young tissues the presence of few senescent cells would permit the turnover of damaged or lost cells by a transient SASP response. This, could lead to temporary cell reprogramming and subsequent proliferation/differentiation. Opposite, the chronic release of SASP factors in aged tissue, due to senescent cell accumulation, would effectively make this cell reprogramming permanent. As a consequence, this would keep the neighboring recipient cells locked in this stem-like state, affecting replacement of lost cells in aged tissue [236].

### *Tissues of BubR1 deficient mice are characterized by senescent hepatocytes/SASP increase*

A further indication of the possible involvement of SASP in the impairment of tissue regeneration in elderly, stems from a previous study of Baker et al. [50]. In this study it was shown accumulation of p16INK4a positive senescent cells and increased SASP in fat and muscle tissues of BubR1 deficient mice [50] which develop early ageing-associated phenotypes [49–53]. In the same study, deletion of p16 coding gene was found to be associated to increased cell replication and decreased SASP. Furthermore, a later study found that clearance of senescent cells in tissues of BubR1 insufficient mice was able to prevent or delay tissue dysfunction and to extend health span [238]. Since BubR1 deficiency is involved in age-dependent impaired liver regeneration [25], collectively, these findings suggest a possible role for SASP in the reduced liver regenerative response.

### *Autophagy reduction, cell senescence and impairment of regeneration in aged livers*

The major inducible pathways for degradation of cellular constituents in eukaryotic cells is represented by autophagy. Autophagy plays an essential role in cellular metabolism and homeostasis by degrading both long-lived cytoplasmic proteins and dysfunctional organelles via lysosome-dependent machinery [239,240]. Recently, the potential involvement of autophagy in aging and aging-associated organ injuries has become evident. Accordingly, to Baker’s study [238], defective autophagy reduces the lifespan, whereas induction of autophagy has been associated to increased longevity in multiple animal species [241,242]. Furthermore, it has been demonstrated that aged livers are characterized by a decline in autophagy activity and that its restoration is able to improve cellular maintenance and hepatic function [243,244].

Recently, Liu et al. [245] showed that plasma from young mice attenuates hepatic injury and restores liver regeneration capacity after PH in aged animals. Indeed, reduction of endoplasmic reticulum stress, hepatocellular damage and autophagosomes and an increase of proliferating cells were observed in aged livers treated with young plasma. Intriguingly, this effect was abolished under administration of autophagy inhibitors. This finding has been further consolidated by *in vitro* data showing that young serum prevented old hepatocytes from senescence and that its effect was abrogated by autophagy inhibition [245]. Based on these observations, the Authors concluded that restoration of autophagy could inhibit cellular senescence supporting a direct link between senescence, autophagy and liver regeneration. Indeed, since senescent cells alter tissue structure and function through SASP release, their elimination by autophagy could alleviate aging-induced hepatic injury and restore the regenerative response.

## CONCLUSION

The long standing concept that hepatocytes lose their proliferative capacity with ageing has been challenged by several experimental evidences based on a successful expansion of hepatocyes even after several rounds of transplantation [212]. Remarkably, aged hepatocytes also retain their fully proliferative capacity if exposed to the treatment with direct mitogenic stimuli, such as ligands of the nuclear receptor CAR, which do not cause liver injury [81].

More recently, increasing evidence suggests that the age-dependent decline of the liver regeneration capacity is the consequence of multiple intertwining factors, both intra and extra-cellular, that cooperate to affect liver mass recovery after tissue damage. From the analysis of the latest literature reports, it emerges that the mass recovery of the injured liver in aged animals is compromised by at least three factors:

i) decreased expression of cell adhesion proteins leading to weakened microstructural adaptation after tissue injury and p21-dependent cell cycle arrest [46];

ii) change of HSC morphology which results in reduced liver perfusion and, consequently, leads to an impairment of tissue reconstitution after damage [196];

iii) chronic release of stemness-inducing pro-inflammatory proteins by senescent hepatocytes, which accumulate in the elderly due to a decline of the autophagy program [235,245].

Thus, while no major age-dependent changes in the expression/activation of factors classically associated to liver regeneration (IEGs, genes coding for cytokines/growth factors or transcription factors) have been convincingly demonstrated [45], it is becoming evident that the altered expression/function of genes (BubR1, YAP, SIRT-1) and cells (HSCs) directly or indirectly involved in injured tissue reconstitution are responsible for the impairment of liver regeneration during ageing [46,57,144,145,196]. Such an hypothesis also explains why no difference in the proliferative capacity of aged and young hepatocytes can be observed following administration of mitogenic agents devoid of hepatotoxicity [81].

An additional cause of the age-dependent decline of hepatocyte proliferation has been identified in the senescent hepatocyte-induced SASP release. Indeed, SASP accumulate during ageing consequently to a decline of the autophagy program; SASP accumulation, in turn, maintains the neighboring recipient cells locked in a stem like state in aged tissues, affecting their capacity to replace lost cells [236].

Age-dependent decreased expression of genes regulating cell adhesion as well as SASP secretion by senescent cells might also be dictated by changes in circadian rhythms occurring during aging [131,145,236], adding further complexity to this topic.

On these bases, a potential therapeutic approach of direct mitogens to relieve the proliferative decline taking place in aged injured liver could be proposed [24]. Treatment with nuclear receptor ligands could also be useful in liver transplantation and hepatic failure in order to restore liver function. Furthermore, therapeutic interventions aimed at eliminating senescent cells or blocking their effects may be useful to treat or delay age-related diseases [236]. In this regard, it also would be interesting to evaluate if ligands of nuclear receptors could have a role in this process. Indeed, as nuclear receptors are ligand-induced transcription factors [246], their activation could unlock SASP-mediated senescence-stem locked cells by reprogramming their gene expression thus eliciting a similar hepatocyte proliferation response in young and aged livers.

In conclusion, in view of the many still unanswered questions, a greater understanding of the molecular mechanisms responsible for the impairment of the regenerative response of the liver is both a priority and a fascinating scientific challenge that may promote the development of innovative concepts that can be translated into the clinic.
